# Analysis of the characteristics of intestinal microbiota after oral tolerance in infants with food protein–induced proctocolitis

**DOI:** 10.3389/fped.2024.1338294

**Published:** 2024-04-26

**Authors:** Xiong Lijing, Zhou Mengyao, Li Jing, Li Yang, Xie Xiaoli

**Affiliations:** Department of Pediatric Gastroenterology, Chengdu Women’s and Children’s Central Hospital, School of Medicine, University of Electronic Science and Technology of China, Chengdu, China

**Keywords:** food protein-induced proctocolitis, intestinal microbiota, amino acid-based formula, oral tolerance, infants

## Abstract

**Objective:**

To understand the characteristics of the intestinal microbiota after oral tolerance in infants with food protein–induced proctocolitis (FPIAP) treated with amino acid formula and their differences from healthy children, aiming to provide a scientific basis for guiding the application of probiotics during treatment.

**Methods:**

FPIAP infants were prospectively enrolled, fecal specimens were obtained, and DNA was extracted for PCR amplification of the bacterial 16S rRNA gene V4 region. Library construction and sequencing were performed, and bioinformatic analysis was performed after obtaining valid data.

**Results:**

There were 36 patients in the FPIAP group: 20 males and 16 females, age 21.944 ± 13.277 months. Diarrhea with blood in the stool were the main symptom, with an average course of 14.83 ± 9.33 days. Thirty infants (83.33%) had mucus stool, 11.11% (4/36) of them experiencing vomiting, and 55.56% (20/36) of the infants displaying poor intake and weight gain, 28 (77.78%) patients with moderate eczema, 2 (5.6%) patients with chronic respiratory symptoms. The treatment time with amino acid formula was 5.51 ± 2.88 months. A control group comprising of 25 healthy infants who were full-term, natural delivery, bottle fed, and matched in terms of age (24.840 ± 12.680 months) and gender (15 males and 10 females) was selected. Anaerobic bacteria were less abundant in FPIAP infants than healthy infants (*P* = 4.811 × 10^−5^), but potentially pathogenic bacteria were more abundant (*P* = 0.000). The abundance of Actinobacteria was low in FPIAP infants, the abundance of *Proteobacteria* was high, and the abundance of *Firmicutes* was reduced. *Bifidobacterium* could be used as a bacterial genus to differentiate healthy and FPIAP infants. Both α-and β-diversity indicators of intestinal microbiota were lower in FPIAP infants. In FPIAP infants, glucose and energy metabolism and amino acid anabolism were decreased, and inflammation-related lipopolysaccharide synthesis pathways were increased.

**Conclusion:**

Compared with healthy infants, FPIAP infants with oral tolerance after amino acid formula treatment had differences in the structure and diversity of intestinal microbiota, among which *Bifidobacterium* was significantly reduced.

**Trial Registration:**

This trial was registered on https://register.clinicaltrials.gov/.

## 1 Introduction

Infants are still establishing their intestinal microecology, their immune system is immature, and their intestinal barrier function is not perfect. Therefore, the immune system of infants is more prone to allergic reactions in early life. Colonization of the intestinal microbiota acts as an antigen that activates gut defense mechanisms, helps the gut immune system develop, establishes oral tolerance, and alters the body's response to potential allergens (1). The incidence of non-IgE-mediated gastrointestinal food-induced allergic disorders (non-IgE-GI-FAs) is increasing yearly (2). Food protein–induced allergic proctocolitis (FPIAP), which is mainly manifested by blood in the infant's stool, is the most common -IgE-GI-FA seen in clinical practice (3). Allergen avoidance is the most important method for the treatment of such diseases, among which amino acid formula is an effective special medical formula for the treatment of infants with FPIAP (4, 5).

Little research has been done on the changes in the intestinal microbiota of infants treated with special medical formulas. Therefore, the purpose of this paper is to clarify the characteristics of the intestinal microbiota of FPIAP infants with oral tolerance established after treatment with amino acid formula and their differences from healthy infants with the same feeding method. Our findings provide a scientific basis for guiding the application of probiotics in the FPIAP treatment process.

## 2 Methods

### 2.1 Subjects

The subjects of the study were infants who were diagnosed with FPIAP. The specific inclusion criteria were (1) age at diagnosis and initiation of treatment were 1–12 months; (2) oral tolerance confirmed by challenge after treatment with amino acid formula; and (3) informed consent of the parents. Exclusion criteria: (1) premature infants; (2) severe malnutrition; (3) complicated intestinal bacterial infection; (4) antibiotics used within 2 weeks; (5) probiotics used for more than 2 weeks; (6) congenital gastrointestinal malformations; (7) history of intestinal surgery; (8) presence of other diseases, such as genetic metabolic diseases.

The diagnosis of FPIAP was confirmed by open oral food challenges (OFCs). Infants presenting with typical clinical symptoms and suspected of having FPIAP underwent a minimum of 2 weeks on an elimination diet prior to the challenge. If the symptoms were resolved, OFCs were conducted to establish the diagnosis. The enrolled infants previously diagnosed with FPIAP received amino acid for a period of time, and then open OFCs were performed to estimate the oral tolerance after the treatment.

To rule out a false-positive challenge due to lactose intolerance, the challenge procedure was performed with lactose-free infant formula. The procedure of OFC referred to the ESPGHAN practical guideline of diagnostic approach and management of CMPA in infants and children4. Firstly, lactose-free CMP-containing infant formula was dropped on the lips. If there was no reaction after 20 min, the formula was given orally and the dose was increased step-wise (1, 3, 10, 30, 100 ml) every 20 min. After the maximum dose was consumed, additional observation under medical supervision could be conducted in hospital for at least 4 h. If no symptoms appeared, the infant would drink at least 200 ml of the lactose-free infant formula every day during the following 2 weeks. Parents were asked to report and record the symptoms with severity occurring at home, then, took infants back to hospital for follow-up the next day. When the symptoms occurred and were considered as positive during the process of provocations, open OFCs were terminated.

### 2.2 Study design

This was a prospective cohort study. Infants with FPIAP who were treated with amino acid formula powder without added probiotics or prebiotics were recruited. After the AAF treatment, the oral food challenges (OFCs) were used to determine the establishments of oral tolerance. Then, the fecal specimens were obtained. At the same time, healthy infants matched for age, gender, feeding method, and delivery method were selected as the control group. Fecal specimens in the sample treatment solution for high-throughput 16S rRNA sequencing detection of intestinal microbiota. All the infants were from the Han ethnic group in Chengdu, the central city located in west China. This study was conducted in accordance with the approved protocol by the Ethics Committee of Chengdu Women's and Children's Central Hospital.

### 2.3 Estimation of sample size

Taking the ratio of *Firmicutes/Bacteroidetes* as the main indicator, according to the literature reports and preliminary experimental results, and setting α* *=* *0.05 (bilateral), 1−β = 0.90, effect size h = 0.80, and 1:1 group ratio, the sample size calculated by PASS 15 software was 20 patients in each group. Assuming a 20% dropout rate, at least 24 patients in each group were needed.

### 2.4 Specimen detection and analysis

The sodium dodecyl sulfate lysate freeze‒thaw method was used for DNA extraction. The bacterial 16S rRNA gene V4 region was amplified by PCR, a library was constructed, and the Illumina NovoSeq6000 platform was used for sequencing. After reading the sequence information, quality control and filtration were performed. Sequence data analyses were mainly performed using QIIME2 and R packages (v3.2.0). OTU-level ranked abundance curves were generated to compare the richness and evenness of OTUs among samples. Taxa abundances at the phylum, class, order, family, genus and species levels were statistically compared among samples or groups by Kruskal.test. OTU-level alpha diversity indices, such as Chao1 richness estimator, Shannon diversity index, and Simpson index, were calculate. Beta diversity analysis was performed to investigate the structural variation of microbial communities across samples using UniFrac distance metrics and visualized via principal coordinate analysis (PCoA). Differences in the Unifrac distances for pairwise comparisons among groups were determined using Student’s t-test and the Monte Carlo permutation test with 1,000 permutations. LEfSe (Linear discriminant analysis effect size) was performed to detect differentially abundant taxa across groups using the default parameters. Random forest analysis was applied to discriminating the samples from different groups using the R package “randomForest” with 1,000 trees and all default settings. Microbial functions were predicted by PICRUSt 2 (Phylogenetic investigation of communities by reconstruction of unobserved states, https://github.com/picrust/picrust2/). Bacterial functions were also analyzed using human gut metabolic modules database. For each sample, functions were profiling by using the Omixer-RPM version 1.0 (https://github.com/raeslab/omixer-rpm) with Kyoto Encyclopedia of Genes and Genomes (KEGG) metabolic pathway predicted from PICRUSt 2. The output file was further analysed using Statistical Analysis of Metagenomic Profiles (STAMP) software package.

### 2.5 Statistical analysis

Clinical data were analyzed with SPSS 22.0 software. Categorical data were compared using the chi-squared test or Fisher's exact test. Continuous variables with a normal distribution are expressed as mean ± variance, and Student's t test was used to compare them; continuous variables without a normal distribution are expressed as median, and the Mann–Whitney nonparametric test was used for comparison. When *P* < 0.05, a variable was deemed significantly different between groups.

To obtain the metabolic pathway results, microbial functions were predicted by PICRUSt 2 (Phylogenetic investigation of communities by reconstruction of unobserved states, https://github.com/picrust/picrust2/), based on high-quality sequences. Bacterial functions were also analyzed using human gut metabolic modules database and gut–brain modules. For each sample, functions were profiling by using the Omixer-RPM version 1.0 (https://github.com/raeslab/omixer-rpm) with KO redundants predicted from PICRUSt 2. The output file was further analysed using Statistical Analysis of Metagenomic Profiles (STAMP) software package v2.1.3.

## 3 Results

### 3.1 Clinical characteristics

A total of 36 patients (20 males and 16 females) were included in the FPIAP group. Their average age was 298.83 ± 155.02 days. After undergoing AAF treatment, all children successfully passed the oral food challenge (OFC), indicating their achievement of oral tolerance. There were a total of 25 infants in the healthy control group. Diarrhea with blood in the stool was the main symptom in FPIAP infants, with an average course of 14.83 ± 9.33 days. The FPIAP group had 28 (77.78%) patients with eczema, 2 (5.6%) patients with chronic respiratory symptoms, and no infants with malnutrition. The treatment time with amino acid formula was 5.51 ± 2.88 months. A control group comprising of 25 healthy infants who were full-term, natural delivery, bottle fed, and matched in terms of age (24.840 ± 12.680 months) and gender (15 males and 10 females) was selected.

### 3.2 Comparison of the composition of the intestinal microbiota

**Figure 1:**
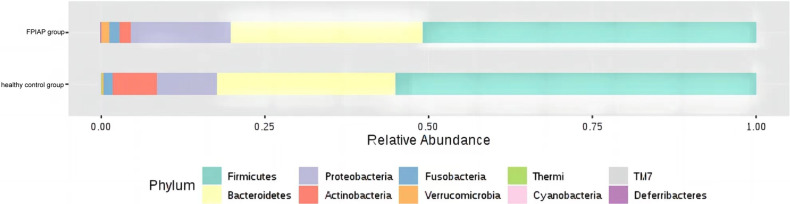
Differences in relative abundance at the level of the FPIAP infant gut microbiota phylum.

**Figure 2:**
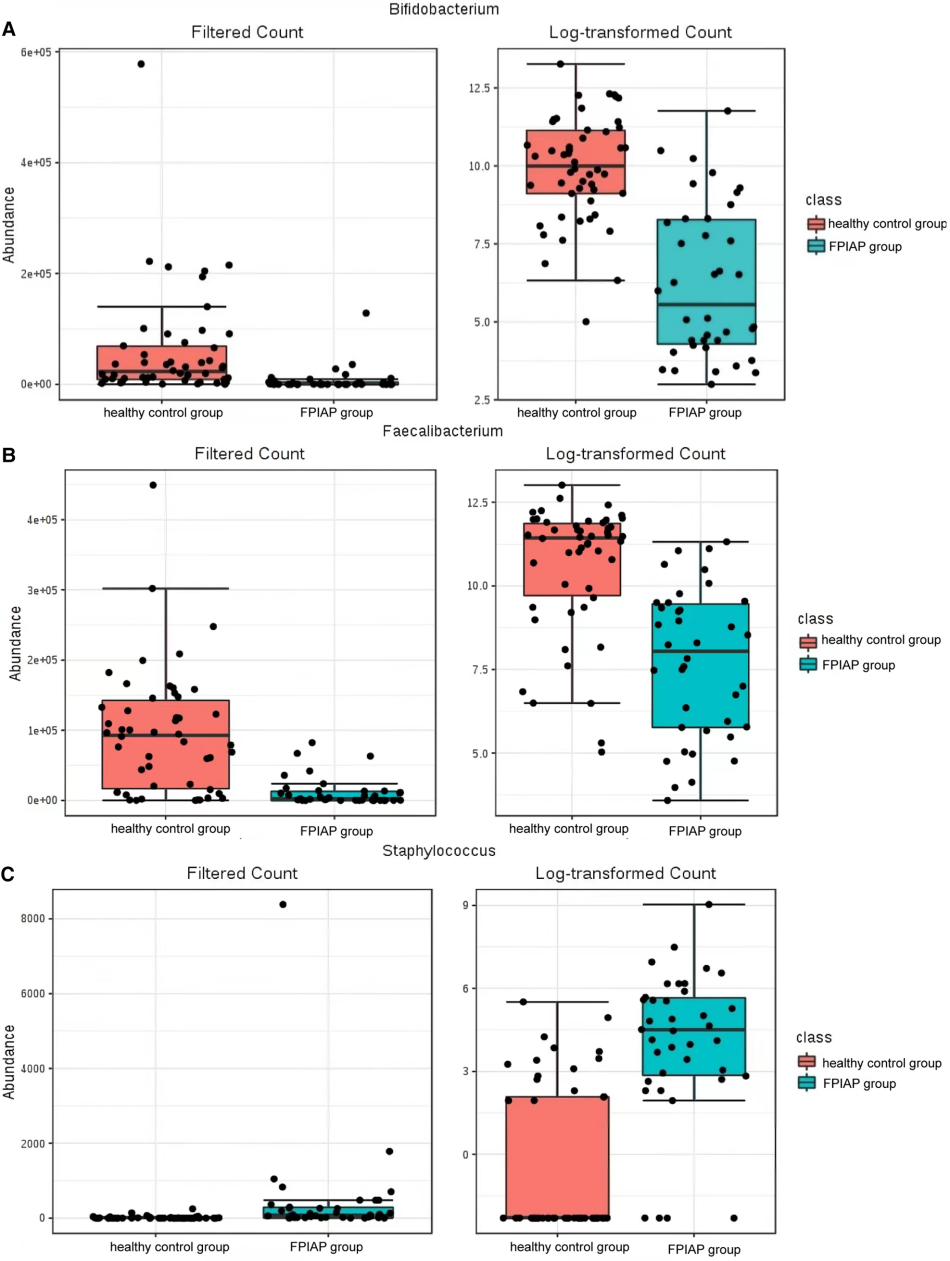
(**A**) *Bifidobacterium* bifidum (**B**) *Bacillus faecalis* (**C**) Staphylococcus.

There were some differences in the overall structure of the intestinal microbiota between the two groups of infants. Anaerobic bacteria were less abundant in FPIAP infants than healthy infants (*P* = 4.811 × 10^−5^), while potential pathogenic bacteria were more abundant (*P* = 0.000). Comparison of the intestinal microbiota of the two groups of infants at the phylum level showed that Actinobacteria was significantly less abundant in FPIAP infants than the control group. *Proteobacteria* was more abundant, *Firmicutes* was less abundant, and there was little difference in *Bacteroidetes* or Fusobacterium between the two groups (Figure 1). Comparison at the genus level showed that the abundance of *Bifidobacterium* was significantly decreased in the intestinal microbiota of FPIAP infants (Figure 2A). FPIAP infants had a lower level of *Bacillus faecalis* and a higher level of *Staphylococcus* (Figures 2B,C).

### 3.3 Microbial diversity analysis

**Figure 3:**
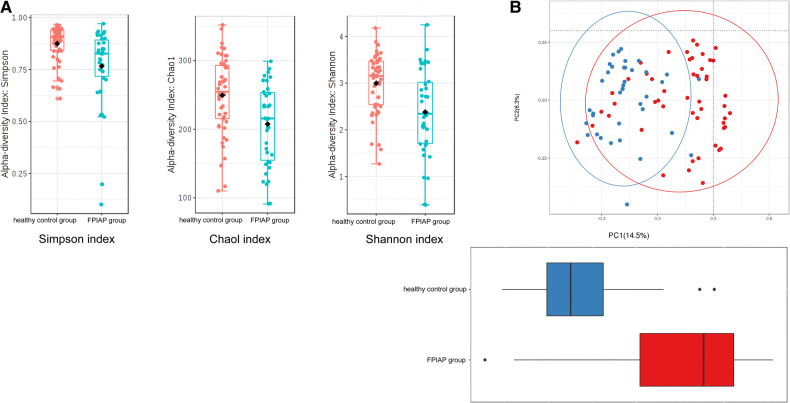
(**A**) α-diversity (**B**) β-diversity.

α-Diversity analysis showed that the Simpson index (*P* = 0.002), Shannon index (*P* = 0.0002), and Chao1 index (*P* = 0.0012) of the intestinal microbiota of FPIAP infants were all decreased (Figure 3A). β-Diversity was also decreased (*P* < 0.001) (Figure 3B). These findings indicate that the diversity of the intestinal microbiota of FPIAP infants was lower than that of healthy infants.

### 3.4 Differential microbiota analysis

**Figure 4:**
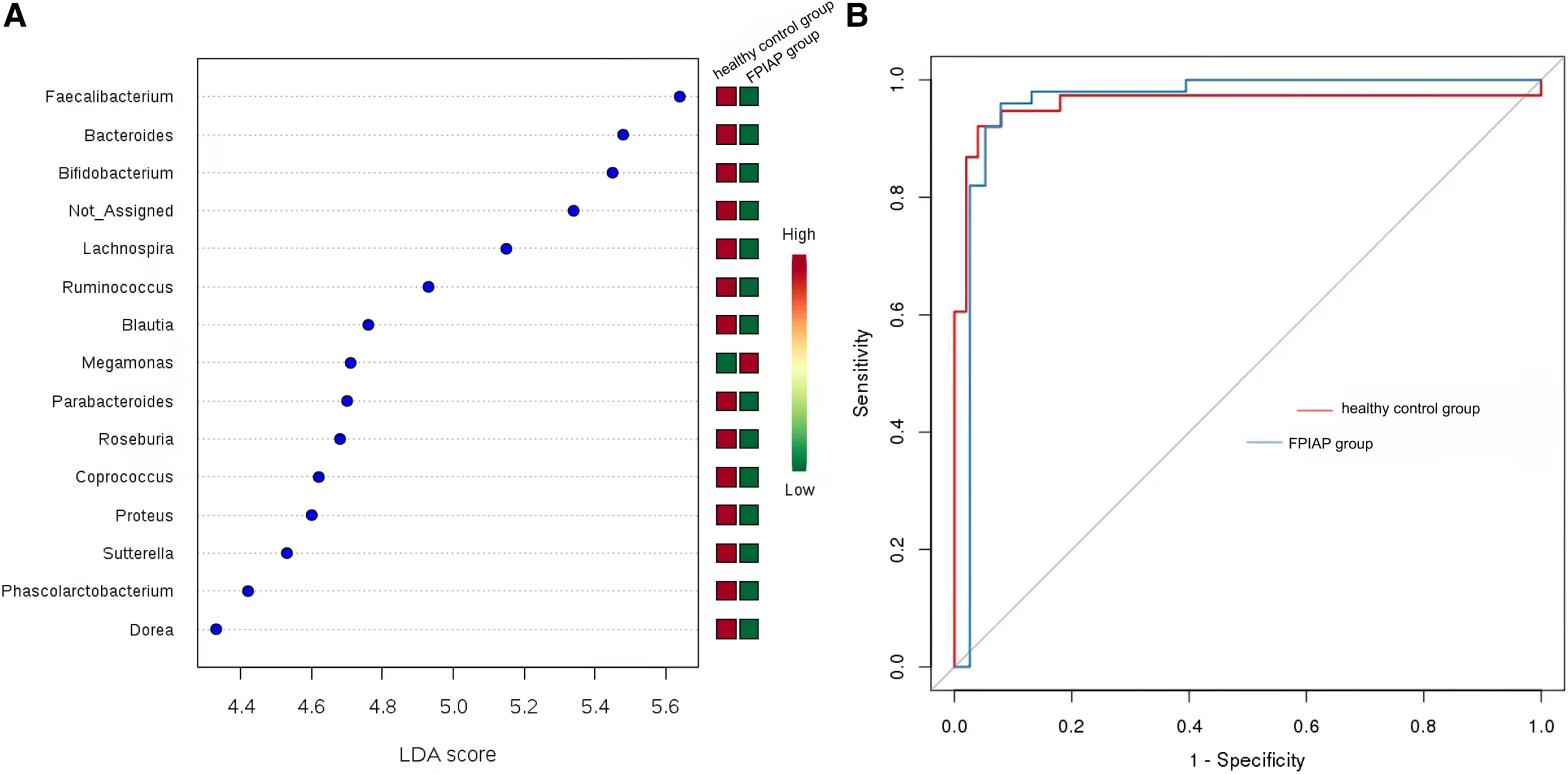
(**A**) LDA score (**B**) ROC curve.

LEfSe analysis screened out landmark communities with LDA score >2. *Bacillus faecalis*, Bacteroides, and *Bifidobacterium* were significantly higher in healthy children than in FPIAP infants, but some other common symbiotic bacteria decreased in the FPIAP group. *Megamonas* was significantly lower in the FPIAP group than in the healthy group. Random forest analysis suggested that *Bifidobacterium* could be used as a characteristic differential genus to distinguish healthy infants from FPIAP infants. The receiver operating characteristic (ROC) curve suggested its high sensitivity (AUC = 0.96) (Figures 4A,B).

### 3.5 KEGG metabolic pathway analysis

**Figure 5:**
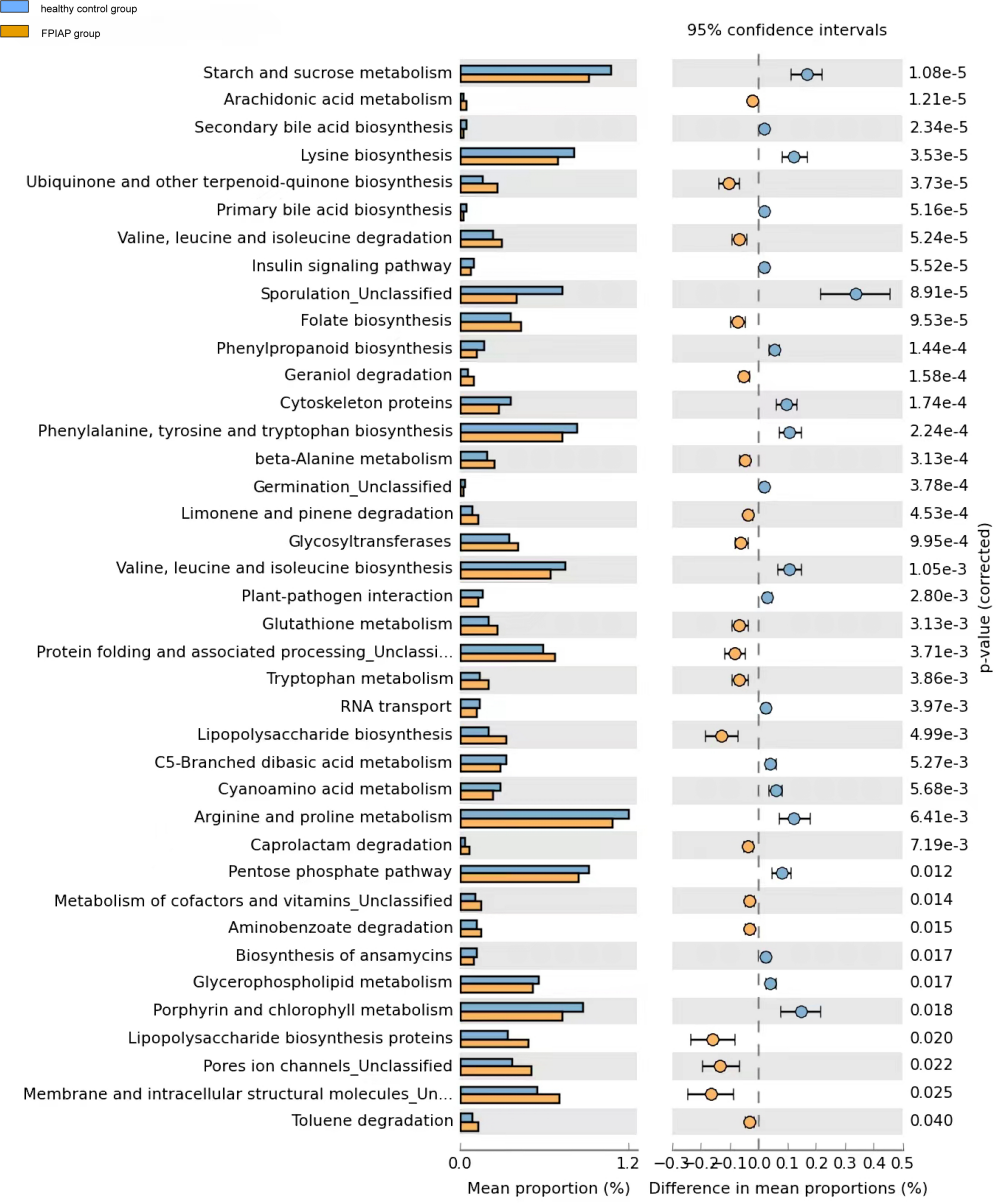
KEGG metabolic pathway.

Compared with healthy infants, FPIAP infants showed decreased sugar and energy metabolism (starch and sucrose metabolism, pentose phosphate pathway, glycerophospholipid metabolism) and amino acid metabolism (lysine, phenylalanine, tyrosine, tryptophan, valine, leucine, isoleucine, arginine, glutathione and proline synthesis), while inflammation-related lipopolysaccharide synthesis pathways were upregulated (Figure 5).Besides, cellular processes included membrane and intracellular structural molecules, pores ion channels, protein folding and associated processing were increased in FPIAP infants.

## 4 Discussion

The intestinal microbiota is important in non-IgE-GI-FAs. Since the intestinal microbiota is involved in the development of intestinal immunity, it is also naturally related to the establishment of oral tolerance. Amino acid formulas are one of the most clinically used special medical formulas since they do not have food allergens and quickly relieve the clinical symptoms of FPIAP. A few studies have found changes in the intestinal microbiota of allergic infants during treatment. Some scholars carried out bacterial culture on the feces of allergic infants and found that after 6 months of oral administration of extensively hydrolyzed formula (eHF), the proportion of Lactobacillus in the intestinal microbiota increased, while the proportions of *Enterobacter* and *Bifidobacterium* was decreased (6). Later, Diaz M et al. found that the proportion of *Bacteroidetes* decreased after a restricted milk diet in infants with non-IgE-mediated allergies (7). However, with the development of the immune system of FPIAP infants, when oral tolerance is finally achieved after long-term use of amino acid formula, it is not well known how the intestinal microbiota of FPIAP infants differs from that of healthy infants.

This study found that compared with healthy infants with the same feeding method, the diversity of the intestinal microbiota, including α-diversity and β-diversity, decreased to varying degrees in FPIAP infants who achieved oral tolerance after a period of amino acid formula treatment. Second, the relative abundance of Actinomycetes, *Proteobacteria*, and *Firmicutes* at the phylum level was different between the two groups in terms of intestinal microbiota structure. FPIAP infants had increased *Proteobacteria* and decreased *Actinobacterial* abundance. *Verrucomicrobia* was found in them, which is not commonly found in the intestinal microbiota of healthy infants, but its significance is unclear. The *Bifidobacterium* in the intestinal microbiota of FPIAP infants treated with amino acid formula was significantly reduced, and the reduction of this genus was a characteristic feature of this group, so it can be highly sensitive at distinguishing allergic infants. Previous studies showed that cow's milk protein allergy infants is associated with dysbiosis of the intestinal microbiota, characterized by a reduction in *Bifidobacteria* and an increase in *Proteobacteria*. Furthermore, cessation of breastfeeding exacerbates this process (3, 8–11). Our study showed that even if oral tolerance were achieved after treatment, the intestinal tract of these infants still lacked important intestinal autochthonous microbiota. The absence of *Bifidobacteria* during infancy and the excessive proliferation of *Proteobacteria* are believed to confer a predisposition to asthma and allergies in later childhood (12–14).Two longitudinal studies were conducted to investigate the composition of early-life gut microbiota in infants with FPIAP (15) and in children with food allergy (16), revealing stable diversity but notable shifts in microbiota abundance. Specifically, there were observed decreases in the abundance of Bifidobacterium and Clostridium. It suggests the presence of intestinal dysbiosis associated with the theory of allergic march. The study conducted by Martin VM et al (15). observed a significant increase in the abundance of *Lactobacillus* in infants with resolved symptoms of FPIAP, which was different from our findings. It is worth noting that their study included predominantly breastfed children, whereas we specifically selected AAF-fed infants. These discrepant results suggested that different treatment modalities might have varying effects on the gut microbiota in infants with FPIAP.

A study observed that the gut microbiome from infants with cow's milk-induced FPIAP broke the intestinal immune balance in mice, by influencing the intestinal Treg cells and Th2 regulation (17).Therefore, special attention should be given to supplementing such probiotics and prebiotics that promote the growth of autochthonous microbiota during the treatment process to give the intestinal microbiota a healthier structure.

In recent years, some scholars have carried out clinical research on special medical formula powders supplemented with probiotics. Among them, the eHF formula with *Lactobacillus rhamnosus* is considered to promote oral immune tolerance (18), but there are relatively few studies on amino acid formulas. An open-label, nonrandomized, multicenter trial evaluated amino acid formula supplemented with two human milk oligosaccharides (HMOs) for feeding infants with moderate to severe cow’s milk protein allergy. There were significant differences in the intestinal microbiota of allergic infants between before and after feeding: The abundance of *Bifidobacterium* in infants of the HMO group increased, the short-chain fatty acids in the feces increased, and the abundance of *Proteobacteria* increased, indicating that amino acid formula supplemented with HMOs can correct intestinal microbiota dysbiosis in allergic infants (19). One study on amino acid formula supplemented with synbiotics (probiotics + prebiotics) for the treatment of infants with non-IgE-mediated milk protein allergy found that amino acid formulas supplemented with synbiotics (fructose-oligosaccharides, inulin, *Bifidobacterium* breve M-16 V) could more effectively regulate the intestinal microbiota and the activity of metabolites, so that the infants with non-IgE-mediated milk protein allergy had an intestinal proportion of *Bifidobacterium* in the microbiota closer to that of breastfed infants, so it helped improve the diversity and metabolic activity of the microbiota in long-term feeding (20, 21). In addition, the improvement of skin symptoms and ear infection in the experimental group was better than that of the control group (22). Our study found an increased abundance of potentially pathogenic *Staphylococcus* in the intestinal microbiota of FPIAP infants, suggesting the possibility of intestinal infection. Therefore, it is necessary to pay attention to the occurrence of intestinal infection in the course of treatment and follow-up.

Changes in the structure of intestinal microbiota will inevitably lead to changes in intestinal metabolic functions mediated by them. Metabolic pathways related to nutrient digestion in the FPIAP infants decreased, mainly in the form of a decline in carbohydrate and energy metabolism and the downregulation of various essential amino acid synthesis pathways. At the same time, pathways related to mediating inflammatory responses, especially lipopolysaccharide and protein synthesis pathways, were upregulated. The above indicates that FPIAP infants have a lesser ability to digest carbohydrates and amino acids mediated by intestinal microbiota due to long-term amino acid powder feeding, and the pathways involved in mediating inflammatory responses and the increase in secondary products may induce intestinal inflammation, which may lead to chronic intestinal inflammation. Therefore, special attention should be paid to the growth and development status of FPIAP infants. After 6 months of age, FPIAP infants should be fed complementary foods as an important supplement to amino acid formula to promote the healthy development of their intestinal microbiota.

However, the KEGG pathways were inferred from microbiome sequencing data using bioinformatic analysis packages and were not directly measured in the stool specimens form infants. Therefore, the results of sequencing data analysis and inference should be corroborated with the metabolomics test results from samples to validate the conclusions. Meanwhile, the study had several limitations including a small sample size, highly selective study population, and the use of 16s rRNA high-throughput sequencing. In future research, it is recommended to expand the sample size and treatment options while utilizing metagenomic sequencing for a more comprehensive characterization of the microbiota characteristics in similar pediatric populations. Furthermore, the comparison of gut microbiota data before and after treatment was lacking, thus this study solely presents the microbiota profile of AAF-fed FPIAP children upon achieving oral tolerance, without being able to differentiate the contribution of the disease itself and treatment to the observed differences.

This study on the characteristics of the intestinal microbiota of FPIAP infants with oral tolerance treated with amino acid formula found that, although the clinical symptoms were relieved, the structure and diversity of their microbiota were still different from those of healthy infants, suggesting that we need to supplement their intestinal autochthonous microbiota in the process of dietary avoidance treatment, to watch out for intestinal infections, and to feed them the correct complementary foods in an effort to promote the healthy development of their intestinal microbiota.

## Data Availability

The original contributions presented in the study are included in the article/Supplementary Material, further inquiries can be directed to the corresponding author.
